# Comparative In Vitro Evaluation of Commercial Periodontal Gels on Antibacterial, Biocompatibility and Wound Healing Ability

**DOI:** 10.3390/pharmaceutics13091502

**Published:** 2021-09-18

**Authors:** Marta Munar-Bestard, Maria Antonia Llopis-Grimalt, Joana Maria Ramis, Marta Monjo

**Affiliations:** 1 Cell Therapy and Tissue Engineering Group, Department of Fundamental Biology and Health Sciences, Research Institute on Health Sciences (IUNICS), University of the Balearic Islands, Ctra Valldemossa km 7.5, 07122 Palma, Spain; marta.munar@uib.es (M.M.-B.); mantonia.llopis@ucc.ie (M.A.L.-G.); 2 Health Research Institute of the Balearic Islands (IdISBa), 07010 Palma, Spain

**Keywords:** periodontal gel, regeneration, antibacterial activity, chlorhexidine, emdogain, gingival fibroblast, engineered 3D gingival tissue, *Porphyromonas gingivalis*, gingipain acitivity

## Abstract

In the last years, several studies testing commercial periodontal gels that contain chlorhexidine (CHX) or other antibacterial agents, have raised concerns regarding their cytotoxicity in periodontal tissues. We aimed at comparing the biocompatibility but also the efficacy as regards to the antibacterial and wound healing ability of different commercial periodontal gels. In vitro human gingival fibroblasts (GF) and a 3D model of human tissue equivalents of gingiva (GTE) were used under inflammatory conditions to evaluate wound closure, cytotoxicity and gene expression. Antibacterial effects were also investigated on *Porphyromonas gingivalis* growth, viability and gingipain activity. In GF and in the bacterial study, we found cytotoxic effects on GF and a high inhibition on bacterial growth rate in gels containing CHX, asiaticoside, enoxolone, cetylpyridinium chloride, propolis and eugenol. Of the two gels that were non-cytotoxic, Syntoss Biogel (containing chondrontin sulfate) and Emdogain (EMD, containing amelogenin and propylene glycol alginate), EMD showed the best wound closure, with no effect on *P. gingivalis* growth but decreased gingipain activity. On the other hand, Syntoss Biogel reduced viability and gingipain activity of *P. gingivalis*, but lack wound healing capacity. In the 3D GTE, Syntoss Biogel and EMD showed a good biocompatibility. Among all the tested gels, formulations containing CHX, asiaticoside, enoxolone, cetylpyridinium chloride, propolis and eugenol showed high antibacterial effect but also showed high cytotoxicity in eukaryotic cells. EMD was the one with the best biocompatibility and wound healing ability at the conditions tested.

## 1. Introduction

Periodontal disease (PD), which includes gingivitis and periodontitis, is one of the most common human diseases, being severe periodontitis the sixth most prevalent disease in the world [1]. PD is characterized by the infection and inflammation of the gingiva that may lead to its destruction, and in severe cases, to the degradation of the alveolar bone and tooth loss.

The tissue damage induced in the PD is caused directly by certain bacteria present in the subgingival plaque and indirectly by an immune response against these bacteria and inflammation, which is mainly produced by macrophages and lymphocytes through a massive production of different cytokine subtypes, including tumor necrosis factor-alpha (TNF-α), interleukin-1 beta (IL-1β), IL-4, IL-6 and gamma interferon (IFN-γ), contributing to the progression of the disease and the death of periodontal tissues [2,3]. The ideal treatment for PD should eliminate bacterial infection, dampen the inflammatory response and stimulate tissue regeneration, thus allowing bone formation and soft tissue attachment to the tooth. Currently, PD treatments allow active disease to be cured using mechanical and antimicrobial procedures. These procedures manage to stop the inflammation, eliminate bacteria and reduce the possible loss of tissue. However, they do not lead to a complete regeneration of the tissue [2,4].

Marketed treatments today for subgingival irrigation in order to control of microbial biofilms in the PD are mostly gels and/or mouthwashes with antiseptics [4]. The most used antiseptic is chlorhexidine (CHX), a bactericidal agent available in the form of acetate, digluconate or hydrochloride, in different concentrations. It has a symmetrical molecular structure, formed by four chlorophenyl rings and two biguanide groups that are connected by a hexamethylene bridge. The formula most commonly used as a periodontal treatment is CHX digluconate, which is effective against both gram-positive and gram-negative bacteria and fungi, and its antimicrobial effect is based on the guanidium cationic groups present that bind to negatively charged bacterial cell walls, acting both as a bacteriostatic (low concentrations) and as a bactericidal agent (high concentrations) [4,5]. Thus, it has been shown that CHX digluconate effectively reduces bacterial plaque and gingivitis, which may delay subgingival bacterial recolonization. Moreover, CHX digluconate has also the ability to bind both to soft and hard oral tissues, resulting in long-term effectiveness after administration [5,6]. However, some adverse effects have been described for CHX, such as brown staining of teeth, tongue and restorations, alterations in the perception of taste, increased deposition of calculus and the possibility of suffering allergic reactions, which have limited the acceptance of this agent [6]. In addition, the current and potential evidence of the cytotoxicity that CHX presents on human gingival fibroblasts, suggests that it may damage periodontal tissue if used in high concentrations or continuously [7].

Other antibacterial agents used in periodontal gels are: (1) Enoxolone (or 18β-glycyrrhetinic acid), which is a major component of licorice plant with antioxidative, anti-inflammatory and antimicrobial activities [8]; (2) eugenol, that is one of the major constituents of clove oil, also with antioxidant, anti-inflammatory and antimicrobial activities [9]; (3) cetylpyridinium chloride (CPC) that is an amphiphilic quaternary compound that possesses antimicrobial activity [10]; (4) propylene glycol alginate (PGA) that is an alcohol reported to show significant antimicrobial action [11] and (5) propolis that is a resin produced by honeybees by mixing wax, pollen, salivary secretions and collected natural resins, with antioxidant, anti-inflammatory antibacterial activity against a range of pathogens and with regenerative properties [12].

PD has been associated with specific Gram-negative bacteria that play a major role in the initiation and progression of disease, such as *Tannerella forsythia*, *Treponema denticola* and *Porphyromonas gingivalis* (*P. gingivalis*) [13]. Other microorganisms that have been implicated as predominant species in PD are: *Aggregatibacter actinomycetemcomitans*, *Fusobacterium nucleatum*, *Prevotella intermedia*, *Campylobacter rectus*, *Peptostreptococcus migros*, *Eikenella corrodens* [14]. *P. gingivalis* is an anaerobic bacteria, which is considered a key pathogen in the PD, since it modulates the expression of genes and proteins that compromise immune function at the periodontal level [15]. *P. gingivalis* possesses several virulent factors such as lipopolysaccharide (LPS) and fimbriae, which can elicit substantial host response reactions, such as the stimulation of the expression of inflammatory cytokines and chemokines by LPS, playing an important role in the pathogenesis of PD and regulating tissue homeostasis [16].

Besides antibacterial agents, periodontal gels nowadays also include regeneration factors such as: (1) aloe vera that is a well-known plant with positive effects on wound healing and regeneration [17]; (2) chitosan that is a biocompatible marine polysaccharide polymer used in several biomedical applications, including wound healing [18]; (3) hyaluronic acid (HA) that is a naturally occurring linear polysaccharide of the extracellular matrix (ECM) of connective tissues that has been shown to promote tissue regeneration [19]; (4) asiaticoside that is a component extracted from the *Centella asiatica* plant and commonly used as a treatment for wounds and burns [20]; (5) chondoitrin sulfate that is one of the main glycosaminoglycans that compose the ECM and favors wound closure [21]; (6) amelogenin fraction of porcine enamel matrix (Emdogain^®^ or EMD) that stimulate cell-matrix interactions and promote the regeneration of damaged periodontal tissue [22] and (7) antioxidants like vitamin C and E that control oxidative stress in wound healing and play important roles in the acceleration of healing [23].

The main target cells of the regeneration factors included in the periodontal gels are gingival fibroblasts (GF), which are the major constituents of periodontal connective tissue and they maintain gingival tissue integrity by regulating collagen and proteoglycan metabolism [24]. In vitro studies on GF for testing oral treatments have mainly been employed in monolayer cultures. However, two-dimensional cultures (2D) have limitations in terms of their ability to simulate the in vivo situation because these models lack the components of the ECM, they lose cell-to-cell and cell-to-matrix interaction that is important for cell differentiation, proliferation and functions in vivo [15]. For this reason, several three-dimensional (3D) models have been developed in recent years using tissue engineering techniques. These 3D models show a higher degree of complexity and structural homeostasis, similar to what occurs in the natural tissues, than cells grown in 2D. The spatial proximity of the cells in this model allows interactions between adhesion molecules and receptors, enabling cell communication and signaling [15,25].

In the last years, many periodontal gels have been commercialized but many of them have only been partially characterized. There is also a lack of a comparative study evaluating their antibacterial, biocompatibility and regenerative properties. Moreover, there are some issues regarding the biocompatibility of CHX on gingival tissues used in most periodontal gels in the market nowadays. Therefore, due to the lack of comparative and complete studies, the aim of our work was to evaluate different formulations of commercial oral gels containing antibacterial and regenerative components, analyzing the cytotoxic and wound healing ability on both 2D and 3D cell culture model of human tissue equivalents of gingiva under inflammatory conditions. Antibacterial effects of the different gels were also investigated on *P. gingivalis* growth, viability and gingipain activity.

## 2. Materials and Methods

### 2.1. Periodontal Gels Used in the Study

Nine different commercial gels were used in the present study, which in their composition include different antibacterial and regenerative factors among other components (Table 1). In preliminary studies, we tested different concentrations of the gels (2.5; 5; 10; 20; 40%) and it was found that the optimal concentration to perform these tests was 5%. Therefore, for the studies on bacteria and cells, periodontal gels were used at 5% (*v*/*v*) concentration as described in Section 2.2.1 and Section 2.3.2. For the study on 3D tissues, periodontal gels were placed directly onto the tissues as described in Section 2.4.3.

### 2.2. Screening of Antimicrobial Activity

#### 2.2.1. Culture and Proliferation Assay of *P. gingivalis*

*P. gingivalis* (33277TM, ATCC, Manassas, VA, USA) was grown at 37 °C for 24–72 h on complete of brain heart infusion (BHI) medium (Scharlab, Barcelona, Spain), supplemented with 0.5 g/L of L-cysteine hydrochloride (Thermo Fisher Scientific, Waltham, MA, USA), 5.0 mg/L of hemin (Thermo Fisher Scientific) and 1.0 mg/L of vitamin K (Thermo Fisher Scientific) under anaerobic conditions (10% H_2_, 10% CO_2_ and 80% N_2_) achieved with an Oxoid Anaerogen^TM^ sachet (Thermo Fisher Scientific).

For treatments *P. gingivalis* were grown from frozen stocks in complete medium BHI. After an overnight incubation, 1 mL bacterial suspensions (≈3 × 10^8^ bacteria/mL) were incubated with the different commercial oral gels (Table 1) at a 5% (*v*/*v*) concentration on BHI medium for 10 h under anaerobic conditions (10% H_2_, 10% CO_2_ and 80% N_2_), achieved with an Oxoid Anaerogen^TM^ sachet at 37 °C. Bacterial suspension without treatment served as negative control, and CHX at 0.2% served as positive control for bacterial growth inhibition. The optical density (OD) was measured at 600 nm at 0 h and 10 h to determine bacterial proliferation (PowerWave Ht, Biotek instruments, Winooski, VT, USA). Bacterial growth rate (µ) was calculated during the exponential growth phase following the equation ln OD_t_ − ln OD_0_ = µ × (t − t_0_), three different experiments were carried out, with two replicates at each condition in each experiment (*n* = 6). Live/Dead ratio of bacteria was determined using the LIVE/DEAD BacLight bacterial viability kit (Invitrogen, Thermo Fisher Scientific), following the manufacturer’s instructions. Moreover, after 10 h bacterial suspensions of periodontal gels and CHX were serially diluted with Dulbecco’s phosphate buffered saline (PBS) (Biowest, Nuaille, France) and 100μL were plated on BHI agar plates, two plates of each dilution were seeded and two independent experiments were run (*n* = 4). The plates were incubated for 7–10 days under strictly anaerobic conditions. The number of colony forming units (CFU) were recorded.

#### 2.2.2. Gingipain Activity of *P. gingivalis*

Gingipains are cysteine proteinases secreted by *P. gingivalis*, which are related to the virulence of the bacteria since they play a critical role in tissue destruction. To determine the Arg-gingipain proteolytic activity, 10 μL of bacterial suspension with the different treatments, after 10 h of incubation, were mixed with 50 μL of assay buffer (10 mM cysteine- Hydrochloric acid (HCL), 1 M HEPES (Biowest), pH 7.5) in microtiter plates. To each well, 100 μL of substrate solution (0.5 mM benzoyl-arginine *p*-nitroanilide (Sigma-Aldrich, St. Louis, MO, USA), 10 mM cysteine-HCL, 50 mM Tris-HCl, pH 7.5) were added. After incubation for 16 h at 37 °C, the absorption at 405 nm was measured (PowerWave Ht). Three independent experiments were performed, with two replicates at each condition (*n* = 6).

### 2.3. Testing of Periodontal Gels on 2D Culture Model with Human Gingival Fibroblasts under Inflammatory Conditions

#### 2.3.1. ihGF Cell Culture

Immortalized Human Gingival Fibroblasts-hTERT (iHGF) (Applied Biological Materials Inc., Richmond, BC, Canada) were grown at 37 °C in an atmosphere of 5% CO2 using fibroblast medium that consists in Dulbecco’s modified Eagle’s medium (DMEM) low glucose (Biowest)/Ham’s F12 (3/1) (Biowest), supplemented with 10% (*v*/*v*) fetal bovin serum embryonic stem cells tested (FBS) (Biowest), 100 µg/mL penicillin and 100 µg/mL streptomycin (Biowest). The culture medium was renewed twice per week. Cells were seeded in 48-well plates at a density of 2 × 10^4^ cells/well. At confluence, these cells were used for the wound closure assay, cytotoxicity and gene expression analysis.

#### 2.3.2. Wound Closure Assay

Forty-eight hours after seeding iHGF in 48-well plates, the monolayer was scraped with a 10 µL sterile pipette tip in a straight line to create a scratch, and cells were washed with growth medium to remove debris and detached cells. Inflammation was then induced by LPS from *P. gingivalis* (InvivoGen, San Diego, CA, USA) at 1 µg/mL and treated with 9 different commercial oral gels (Table 1) at 5% (*v*/*v*) concentration in fibroblast medium with 50 µg/mL ascorbic acid (Sigma-Aldrich) for 48 h. Fibroblast medium with 50 µg/mL ascorbic acid (Sigma-Aldrich) and without gels served as negative control, and CHX (Abcam, Cambridge, UK) at 0.2% served as positive control. Images of the same areas were taken before treatment and also 24 and 48 h after treatment, using a bright-field inverted microscope (Eclipse TS100, Nikon, Tokyo, Japan). The images were quantitatively analysed with the software ImageJ using the default parameter settings. The wound closure area (%) was defined as: (scratch area at time 0—scratch area at 24 or 48 h)/(scratch area at time 0) × 100. Three independent experiments were performed, with two replicates at each condition (*n* = 6).

#### 2.3.3. Cell Cytotoxicity

To estimate cytotoxicity of the different commercial oral gels, the presence of lactate dehydrogenase (LDH) in culture media 48 h after the pro-inflammatory stimulus with LPS and the treatment with the periodontal gels, was used as an index of cell death. Following the manufacturer’s instructions (Cytotoxicity Detection kit, Roche Diagnostics, Mannheim, Germany), LDH activity was determined spectrophotometrically after 30 min of incubation at room temperature (RT) of 50 µL of culture media and 50 µL of the reaction mixture, by measuring the oxidation of nicotinamide adenine dinucleotide (NADH) at 490 nm in the presence of pyruvate. The results were presented relative to the LDH activity of the media of cells seeded on tissue culture plastic (TCP) without treatment (control negative, 0% of cell death), and on cells grown on TCP treated with 1% Triton X-100 (control positive, 100% of death), using the following equation: Cytotoxicity (%) = [(expected value −control negative)/(control positive−control negative)] × 100. Three independent experiments were performed, with two replicates at each condition (*n* = 6).

#### 2.3.4. Gene Expression by Real-Time RT-PCR

After 48 h of treatment, total RNA was isolated using RNAzol^®^ RT (Molecular Research Center, Cincinnati, OH, USA), according to the manufacturer’s protocol and quantified at 260 nm using a Nanodrop spectrophotometer (NanoDrop Technologies, Wilmington, DE, USA). To obtain cDNA, the same amount of RNA (700 ng) was reverse transcribed at 42 °C for 60 min using High-Capacity RNA-to-cDNA kit (Applied Biosystems, Foster City, CA, USA).

Real-time PCR was performed for three reference genes, glyceraldehyde-3-phosphate dehydrogenase (GAPDH), beta-actin (ACTBL2) and 18S ribosomal RNA (18S rRNA), and several target genes (Table 2).

Real-time RT-PCR was performed in the Lightcycler 480^®^ system using SYBR green detection (Roche Diagnostics). Each reaction contained 7 µL of master mix (Lightcycler 480 SYBR Green I Master, Roche Diagnostics), the sense and the antisense specific primers (0.5 µM) (Table 2), and cDNA sample (3 µL), in a final volume of 10 µL. The amplification program consisted of a preincubation step for denaturation of the template cDNA (5 min, 95 °C), followed by 45 cycles consisting on a denaturation step (10 s, 95 °C), an annealing step (10 s, 60 °C), and an extension step (10 s, 72 °C). After each cycle, fluorescence was measured at 72 °C. Water (Sigma-Aldrich) was used as negative control without cDNA. To allow relative quantification after PCR, standard curves were constructed from standard reactions for each target and reference genes. The crossing point readings for each sample were used to calculate the amount of either the target or the reference relative to a standard curve, using the second derivative maximum method provided by the LightCycler480^®^ analysis software version 1.5 (Roche Diagnostics). All samples were normalized by the mean of the expression levels of reference genes and changes were related to the control groups that were set to 100%. Three independent experiments were performed, with two replicates at each condition (*n* = 6).

### 2.4. Testing of Periodontal Gels on 3D Gingival Tissue Equivalents (GTE)

#### 2.4.1. Cell Culture

Immortalized Human Gingival Keratinocytes (Gie-No3B11, abbreviated as iHGK) (Applied Biological Materials Inc., Richmond, BC, Canada) were grown on tissue culture flask for sensitive adherent cells (Sarstedt, Numbrecht, Germany) at 37 °C in an atmosphere of 5% CO_2_ using keratinocyte medium that consist in DMEM without magnesium and calcium (Gibco, Grand Island, NY, US)/Ham’s F12 (3/1) (Biowest), supplemented with 0.01 mg/mL of insulin (Sigma-Aldrich), 0.4 ng/mL of hydrocortisone (Sigma-Aldrich), 6.7 ng/mL of selenium (Sigma-Aldrich), 0.01 μg/mL of human epithelial growth factor (ThermoFisher Scientific), 1M HEPES-buffer (Biowest), 5.5 μg/mL of transferrin (Sigma-Aldrich), 10–10 M of cholera toxin (Sigma-Aldrich), 2 mM of L-glutamine (Sigma-Aldrich), 5% (*v*/*v*) of FBS embryonic stem cells tested (Biowest) and 100 µg/mL penicillin and 100 µg/mL streptomycin (Biowest). The culture medium was renewed twice per week. Immortalized Human Gingival Fibroblasts-hTERT (iHGF) were cultured as described in Section 2.3.1. Both iHGK and iHGF cultures with 70–80% confluency were used for the construction of GTE as described in the next Section 2.4.2.

#### 2.4.2. Engineering 3D Gingival Tissue Equivalent (GTE)

**Figure 1:**
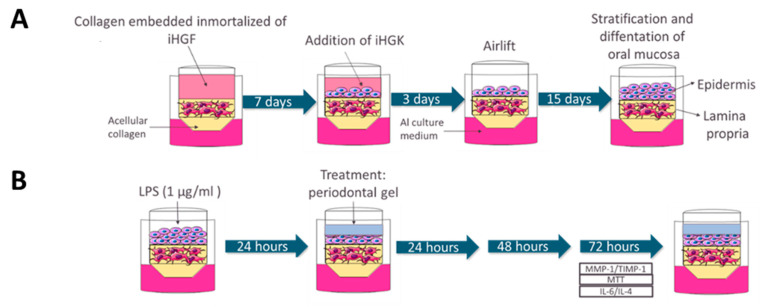
Experimental design for testing periodontal gels on 3D gingival tissue equivalents (GTE). (**A**) Experimental setup 3D of GTE and (**B**) stimulation of GTE with LPS and periodontal gel treatment.

**Figure 2:**
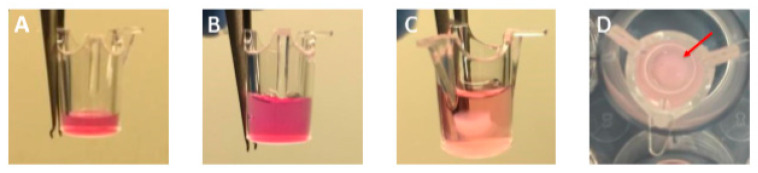
Steps for setting a 3D GTE. The images represent an example of (**A**) acellular collagen; (**B**) collagen-embedded iHGF; (**C**) the mucosal component iHGK addition; (**D**) successful GTE cultivated for 25 days (15 days of airlift).

The GTE was constructed according to the technique described by Dongari Bagtzoglou and Kashleva [26] (Figure 1A and Figure 2). In short, a rat tail type I collagen solution (ThermoFisher Scientific) (2.2 mg/mL) was mixed with iHGF (1 × 10^5^ cells/well) and pipetted into a 24-well transwell insert (Sarstedt) with 0.4 μm pores. The fibroblast-embedded collagen was cultured at 37 °C, 5% CO2 for 7 days submerged in fibroblast medium. iHGK (2.5 × 10^5^ cells/well) were then seeded on top and GTE were cultured submerged in keratinocyte medium for 3 days at 37 °C, 5% CO2. GTE were then lifted to the air–liquid interface and incubated cultures at 37 °C, 5% CO2 for 15–17 days in airlift culture medium (AL) consisting of DMEM low glucose (Biowest)/Ham’s F12 (3/1) (Biowest), supplemented with 5 μg/mL of insulin (Sigma-Aldrich), 0.4 μg/mL of hydrocortisone (Sigma-Aldrich), 2 × 10^–11^ M of 3,3′, 5-triiodo-L-thyronine (T3) (Sigma-Aldrich), 1.8 × 10^–4^ M of adenine (Sigma-Aldrich), 5 μg/mL of transferrin (Sigma-Aldrich), 10^–10^ M of cholera toxin (Sigma-Aldrich, St. Louis, MO, USA), 2 mM of L-glutamine (Sigma-Aldrich), 5% (*v*/*v*) of FBS embryonic stem cells tested (Biowest) and 100 µg/mL of penicillin and 100 µg/mL of streptomycin (Biowest). The AL medium was refreshed every other day. After 25 days GTEs were treated as described in Section 2.4.3 and were harvested for histological analysis as described in Section 2.4.7.

#### 2.4.3. 3D Treatment with Periodontal Gels

At the end of the incubation period of GTE, tissues on the air-liquid interface were treated on top with 50 µL of 1 µg/mL *P. gingivalis* LPS (InvivoGen, San Diego, CA, USA) for 24 h (Figure 1B). Then, 30 µL of Gel H (Syntoss, Israel Ltd., Ashdod, Israel), Gel I (EMD) or PBS (Biowest) for the negative control were applied on top the tissue for 72 h (Figure 1B). Viability assays were performed by MTT test as described in Section 2.4.4. The fibroblast medium outside the insert was changed every day during the treatment, and this culture media was saved for the determination of MMP-1 and TIMP-1 analysed by enzyme linked immunosorbent assay (ELISA) as described in Section 2.4.5, and IL-6 and IL-4 analysed by the multiplex bead immunoassay as described in Section 2.4.6. Experiment was run in six sample replicates (*n* = 6) for each group.

#### 2.4.4. MTT Test

At the end of the incubation period with the periodontal gels, the tissues were rinsed with PBS (Biowest) and placed on 400 μL of 0.5 mg/mL MTT (3-(4,5-dimethylthiazol-2-yl)-2,5-diphenyltetrazolium bromide (ThermoFisher Scientific). After 3 h of incubation at 37 °C and 5% CO2, cultures were placed in 2 mL of isopropanol (Sigma-Aldrich). Extraction was performed overnight at room temperature. Optical density was measured on 200 μL of extracts at 570 nm (reference filter: 690 nm). Negative control was obtained from culture media treated with PBS and was set at 100% each time point. Positive control was obtained from culture media treatment with 10% sodium dodecyl sulfate (SDS) diluted in PBS (1:1). Results are expressed as percentage of viability compared to negative control. Experiment was run in three sample replicates (*n* = 3) for each group.

#### 2.4.5. MMP-1 and TIMP-1

The detection of TIMP-1 and MMP-1 was performed from cell culture media after 72 h of treatment of GTE cultures by commercially available ELISA kits according to the manufacturer instructions (Sigma, St. Louis, MO, USA).

#### 2.4.6. Cytokine Levels

Different pro-inflammatory and anti-inflammatory interleukins were evaluated on GTE culture medium after 72 h of treatment using the multiplex bead immunoassay: IL-6 and IL-4, (Magnetic Luminex Assay, R&D Systems, Minneapolis, MN, USA) according to the manufacturer’s protocols.

#### 2.4.7. Histology and Inmunohistoquemistry

The GTE were fixed in 4% formaldehyde and processed for paraffin embedment. Paraffin sections (6 μm) were cut and were stained with hematoxylin and eosin (H&E) for histological examination or processed for immunohistochemistry (IHC) to study expression of specific proteins. The sections were dewaxed in xylene (30 min) and rehydrated through 100%, 96% and 70% alcohol series for 5 min each. The antigens were retrieved with 0.01 M citrate buffer (pH 6.0) in a microwave (900 W) for 20 min and cooled to room temperature for at least 1 h. Endogenous peroxidase was quenched with 5% H2O2 in water for 10 min and washed in PBS (Biowest). Unspecific proteins were blocked with 2% normal goat serum (NGS, Vector Laboratories, Burlingame, CA, USA) in PBS for 20 min. The sections were then incubated overnight at 4 °C with mouse monoclonal primary antibodies (Table 3). Afterward, the sections were treated with biotinylated anti-mouse secondary antibody (Vector Laboratories) diluted 1:200 for 30 min at room temperature and followed by incubation in avidin-biotinylated peroxidase complex (Vector Laboratories) for 30 min at room temperature. Peroxidase activity was revealed with Sigma Fast 3,3′-diaminobenzidine (Sigma-Aldrich) as substrate for 5 min. Finally, sections were counterstained with hematoxylin and mounted in Eukitt (Kindler, Freiburg, Germany). Images were acquired with a Zeiss Axioskop 2 microscope equipped with AxioCam ICC3 digital camera and AxioVision 40V 4.6.3.0 Software (Carl Zeiss, S.A., Barcelona, Spain).

### 2.5. Statistical Analysis

All data are presented as mean values ± SEM. The Shapiro-Wilk test was done to assume parametric or non-parametric distributions for the normality tests. Differences between groups were assessed by Kruskal-Wallis or by one-way ANOVA with LSD as post-hoc depending on their normal distribution. SPSS^®^ program for Windows, version 17.0 (SPSS Inc, Chicago, IL, USA) and GraphPad Prism (version 7, GraphPad Software Inc., La Jolla, CA, USA) was used. Results were considered statistically significant at *p*-values < 0.05.

## 3. Results

### 3.1. Results of Antimicrobial Activity of Different Periodontal Gels

**Figure 3:**
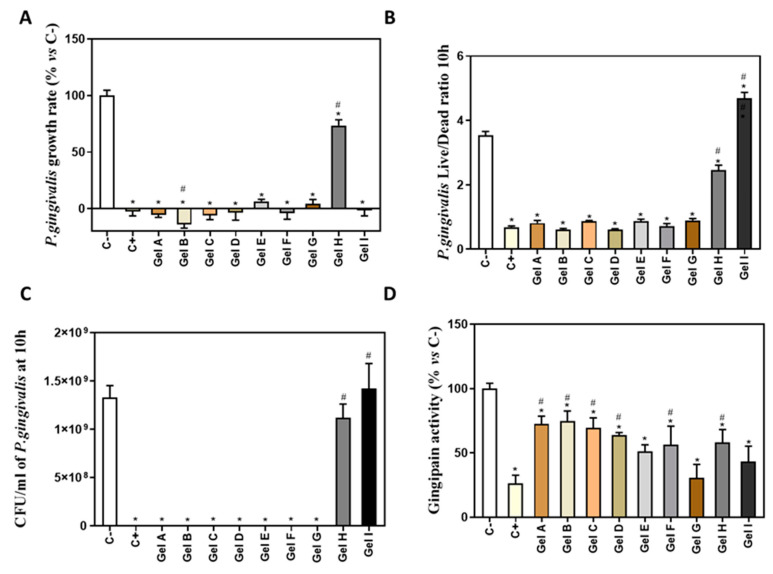
Antimicrobial activity of different periodontal gels. (**A**) *P. gingivalis* growth rate cultured with different periodontal gels (*n* = 6). (**B**) *P. gingivalis* live/dead ratio after treatment for 10 h with different periodontal gels (*n* = 6). (**C**) Number of *P. gingivalis* CFU/mL after 10 h of incubation with the different treatments (*n* = 4). (**D**) In vitro gingipain activity from *P.gingivalis* after 10 h of treatment (*n* = 6). Results are expressed as % vs. Negative control that was set to 100%. Data represent the mean ± SEM. Negative control (C−) was bacterial suspension without any treatment and positive control (C+) was bacterial suspension with CHX at 0.2%. See Table 1 for the identification of gels used in the study. Results were statistically compared by Kruskal-Wallis for *P. gingivalis* growth rate and by ANOVA and LSD as post hoc for *P. gingivalis* live/dead ratio, number of CFU/mL and in vitro gingipain activity: * *p* < 0.05 treatment vs. negative control. # *p* < 0.05 treatment vs. positive control.

To calculate the growth rate of *P.gingivalis* (Figure 3A), the exponential growth phase was set from 0 to 10 h of culture. Gel A, Gel B, Gel C, Gel D, Gel E, Gel F, Gel G and Gel I produced a high inhibition of bacterial growth rate respect to the negative control. Gel H produced a low inhibition of bacterial growth rate, being 74% of growth rate respect to the control.

*P. gingivalis* live/dead ratio after 10 h with different periodontal gels was analyzed to test their effect on bacterial survival. Figure 3B shows a significantly lower live/dead ratio on eight gels (Gel A to Gel H) with respect to negative control, which indicates that they decreased bacterial survival, while Gel I improved viability of bacteria with respect to negative control. Moreover, Gels I and H showed a significantly higher live/dead ratio compared to the positive control, CHX.

As the results obtained for Gel I on the growth rate and the live/dead ratio did not agree with each other, we determined the CFU using the serial dilution method after 10 h of treatment (Figure 3C). These last results were in agreement with the ones obtained on the live/dead ratio, showing no bacterial growth after treatment with gels A to G and a similar growth to the negative control for the groups treated with gels I and H.

We also evaluated the gingipain activity of the bacteria treated with the different periodontal gels (Figure 3D). All gels showed a significantly lower gingipain activity than the negative control. Moreover, Gels A, B, C, D, F, H had a significantly higher gingipain activity than the positive control, CHX.

### 3.2. Effect of Periodontal Gels on 2D Cell Culture

**Figure 4:**
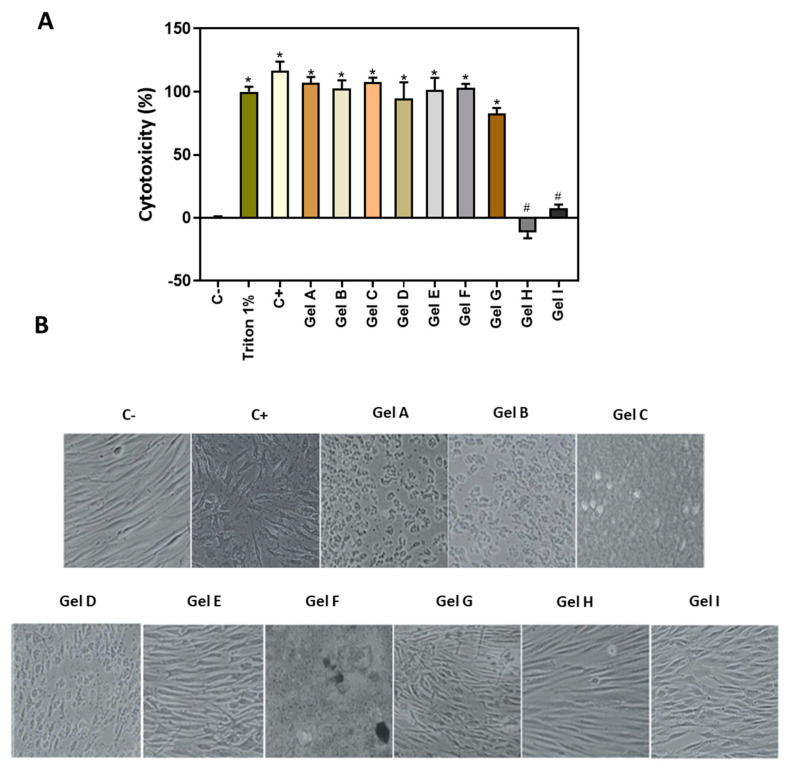
In vitro study 2D cell culture model. (**A**) LDH activity, an indicator of cytotoxicity, measured in culture media after the application of treatments and healing for 48 h. Negative control (C−) (0% toxicity) was obtained from culture media of cells seeded on plastic without treatment. To obtain 100% toxicity, 1% triton X-100 was used. Positive control (C+) was obtained from cells seeded on plastic and treated with CHX at 0.2%. See Table 1 for the identification of gels used in the study. (**B**) Images of cell morphology at 24 h of treatment with the different gels, images were taken at a magnification of 100×. Values represent the mean ± SEM (*n* = 6). Results were statistically compared by ANOVA and LSD as a post hoc: * *p* < 0.05 treatment vs. negative control. # *p* < 0.05 treatment vs. positive control.

LDH assay was used as an indicator of cytotoxicity, as this enzyme leaks out through the plasma membrane of damaged cells. As shown in Figure 4A, the positive control CHX and seven gels (Gel A to Gel G) induced a high release of LDH similar to 1% Triton X-100 that represents 100% cytotoxicity. On the contrary, Gels H and I showed good biocompatibility results on gingival fibroblasts.

Visual observation of the cell morphology after 24 h of treatment (Figure 4B) indicated that cells treated with the positive control and gels A, B, C, D, E, F were toxic, since cells had a round and instead of a fibroblastic shape. In contrast, the negative control cells and those treated with Gels H and I, presented an elongated cell morphology, characteristic of fibroblasts.

**Figure 5:**
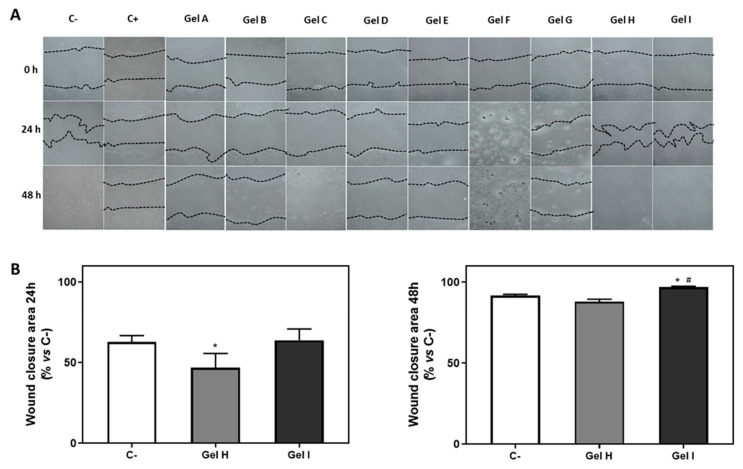
Wound healing assay after periodontal gel treatment. (**A**) Images of wound healing 24 and 48 h after treatment with the different gels, images were taken at a magnification of 100×. (**B**) images of % of wound closure area after 24 h and 48 h of healing in the presence of Gel H or Gel I, in relation to Control. Negative control (C−) was obtained from cells seeded on plastic without treatment and Positive control (C+) was obtained from cells seeded on plastic treated with CHX at 0.2%. See Table 1 for the identification of gels used in the study. Results are expressed as % vs. Negative control that was set to 100%. Values represent the mean ± SEM (*n* = 6). Results were statistically compared by ANOVA and LSD as post hoc: * *p* < 0.05 treatment vs. negative control. # *p* < 0.05 treatment vs. Gel H.

Figure 5A shows representative images of open wound areas at the different time points. While, almost all the wound area was closed after 48 h in negative control and cells treated with Gels H and I, no wound was traced for Gels C and F due to the disruption of the cell monolayer caused by these treatments. The results of the quantification of the % of wound area closed is only shown for gels that presented wound closure after 24 h and 48 h (Figure 5B) with respect to time 0, i.e., for cells treated with gel H and gel I, in addition to the control. After 24 h, a reduction in wound closure was observed for cells treated with gel H, compared to the negative control. On the other hand, after 48 h a higher wound closure was observed for cells treated with Gel I compared to the negative control and Gel H.

**Figure 6:**
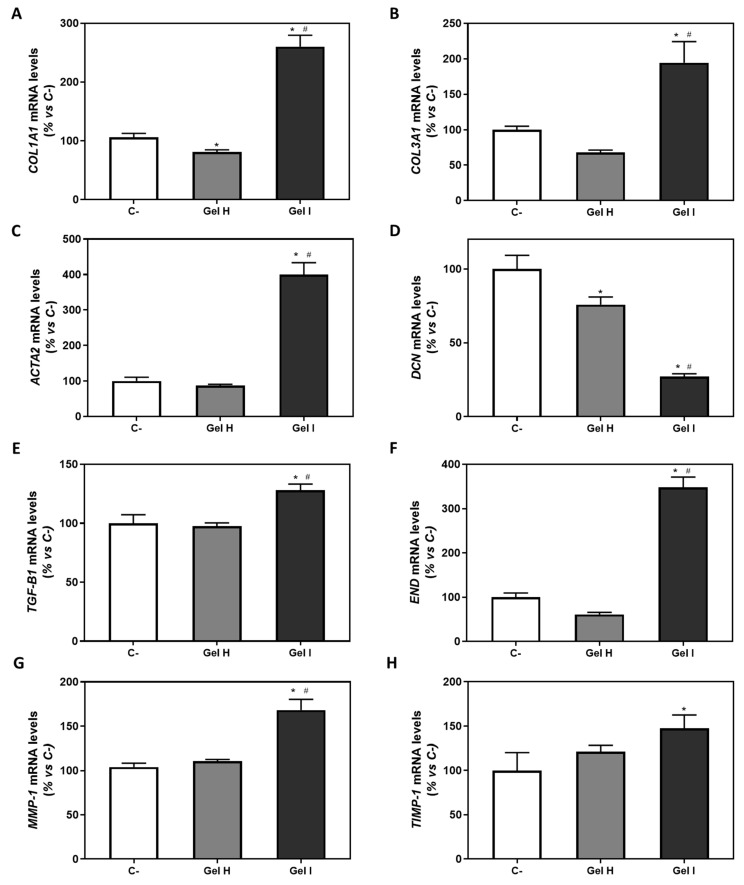
Gene expression levels of marker genes after treatment with periodontal gels. Effect of treatment with (Gel H) and (Gel I) for 48 h on mRNA expression levels of COL1A1 (**A**), COL3A1 (**B**), ACTA2 (**C**), DCN (**D**), TGF-β1 (**E**), END (**F**), MMP-1 (**G**) and TIMP-1 (**H**) in iHGF, in the presence of LPS. Negative control (C−) was obtained from cells seeded on plastic without treatment. See Table 1 for the identification of gels used in the study. Results are expressed as % vs. Negative control that was set to 100%. Values represent the mean ± SEM (*n* = 6). Results were statistically compared by Kruskal-Wallis for Col1A1 and ACTA2; and by ANOVA and LSD as post hoc for Col3A1, TGF-β1, END, DCN, MMP-1 and TIMP-1: * *p* < 0.05 treatment vs. control, # *p* < 0.05 Gel I vs. Gel H.

The effect of the gels on the gene expression levels of different markers of human gingival fibroblasts are shown in Figure 6. Only the biocompatible gels (H and I) were tested compared to the negative control, since the rest of the gels produced cell death and cell monolayer disruption. Cells treated with Gel I showed a significant increase in COL1A1, COL3A1, ACTA2, TGF-β1, END, and MMP-1 mRNA levels with respect to the negative control and to Gel H, and increased TIMP-1 mRNA levels with respect to the negative control. In the case of DCN (Figure 6D), its expression was significantly reduced in the cells treated with Gel I respect to the negative control and to Gel H. On the other hand, cells treated with Gel H showed a significant decrease on COL1A1 (Figure 6A) and DCN mRNA levels compared to the negative control.

### 3.3. Results of Periodontal Gels on 3D Cell Culture Model

**Figure 7:**
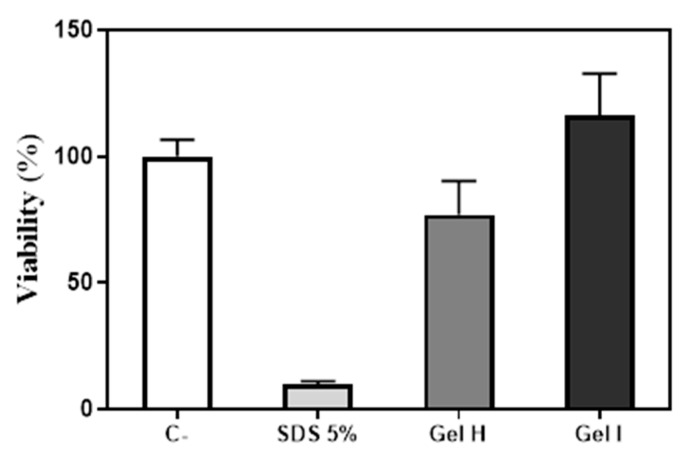
MTT assay on GTE after the application of periodontal gels and LPS for 72 h. Negative control (C−) was obtained from culture media of GTE treated with PBS that was set at 100%. Positive control (SDS 5%) was obtained from culture media of GTE treated with 10% SDS diluted in PBS (1:1). Values represent the mean ± SEM (*n* = 3). Results were statistically compared by Kruskal-Wallis.

The MTT assay was used to evaluate tissue viability after the 72 h of treatment with gels H and I, by measuring the MTT reduction by mitochondrial reductase enzymes. As shown in Figure 7, none of gingival gels tested had a deleterious effect on the viability of GTE. Indeed, the gel I showed greater viability compared to Gel H, although differences did not reach statistical significance.

**Figure 8:**
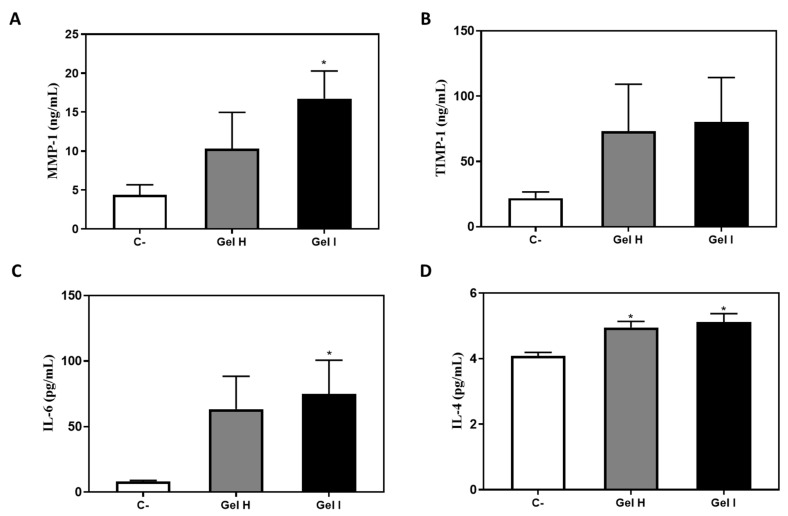
Effect of periodontal gels H and I on MMP-1 (**A**), TIMP-1 (**B**) IL-6 (**C**) and IL-4 (**D**) release by GTE 72 h after treatment and LPS stimulation. Negative control (C−) was obtained from culture media of GTE treated with PBS. Values represent the mean ± SEM (*n* = 6). Results were statistically compared by ANOVA and LSD as post hoc: * *p* < 0.05 treatment vs. negative control.

Production of MMP-1, TIMP-1, IL-6 and IL-4 were increased 72 h after treatments with Gel H and Gel I compared to the negative control (Figure 8), but it reached significant levels for MMP-1, IL-6 and IL-4 for Gel I and for IL-4 for Gel H.

**Figure 9:**
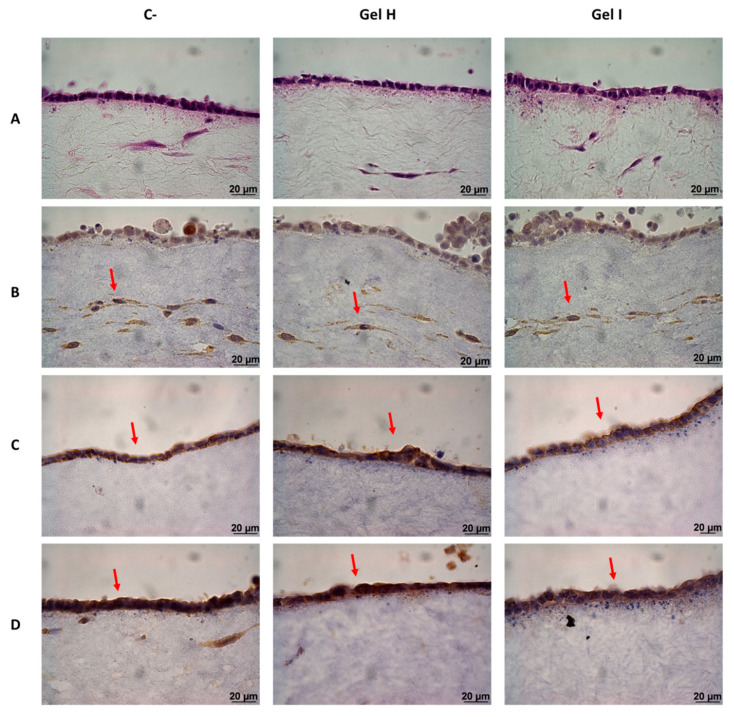

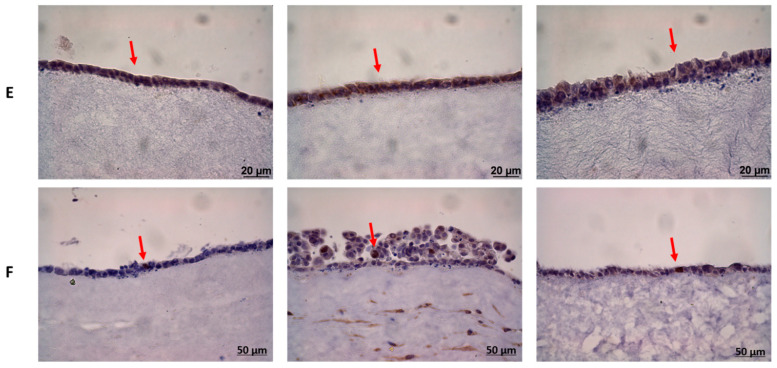
Histologic characterization of GTE after 72 h of treatment with periodontal gels and LPS stimulation. The images represent an example of (**A**) H&E staining of GTE; (**B**) Expression of vimentin (fibroblasts marker); (**C**) Expression of keratin 19 (epithelial differentiation marker); (**D**) expression of keratin 17 (epithelial differentiation marker); (**E**) Expression of involucrin (epithelial differentiation marker); and (**F**) Expression of Ki-67 (proliferation marker), images were taken at a magnification of 630× (Images of (**A**–**E**)) and 400× (Images of (**F**)). Negative control (C−) was obtained from GTE treated with PBS.

GTE was intensively characterized at the histological level (H&E staining and immunohistochemistry (IHC) of epidermal and dermal markers) (Figure 9) after the application of treatments (Gel H and Gel I). H&E staining (Figure 9A) was done to stain nucleus and cytoplasm respectively, images showed a well-defined epidermis on top of a fibroblast matrix, without differences among treatments. The expression of vimentin differentiation marker for fibroblasts (Figure 9B) was also similar in all the groups. As regards to the protein expression of epidermal differentiation markers keratin 19, keratin 17 and involucrin (Figure 9C–E), positive IHC staining was confirmed for all groups in keratynocytes growing in layer. Finally, expression of KI-67 (Figure 9F), a marker of epithelial proliferation at basal layer, was also confirmed in all the evaluated groups.

## 4. Discussion

In this study, we have tested the antimicrobial effect of different marketed periodontal gels using *P. gingivalis* as one of the pathogens of the periodontal disease, and the cytotoxicity and wound healing ability of these gels using a conventional 2D in vitro model with gingival fibroblasts and a novel 3D engineered gingival tissue model to simulate the in vivo situation. We show that most commercial periodontal gels containing CHX or other antibacterial factors exhibited high cytotoxicity effect on gingival cells and high inhibition of bacterial growth rate, while other two periodontal gels showed good biocompatibility on gingival cells and 3D gingival tissue, with more limited antibacterial activity, and only EMD showed wound healing ability (Table 4).

Gel A (Bexident Post Gel), Gel B (Perio-Aid Gel Bioadhesive), Gel D (Clorhexidine Lacer Gel Bioadhesive) and Gel E (Oralsan Gel Gengivale) contain CHX in a high concentration (0.2–0.5%), and they were highly cytotoxic on 2D cultured gingival fibroblasts and exhibited high inhibition of *P. gingivalis* growth rate. This observation is in agreement with the findings observed with the reported effects of CHX, showing strong cytotoxic effect and altered cell morphology on fibroblasts [7] and a strong antimicrobial action [27]. Furthermore, it was verified that, although some of these gels have components that promote wound healing, such as chitosan (Gel A) [18], hyaluronic acid (Gel B) [19] or aloe vera (Gel E) [17], they did not show any regenerative effect on the cells by analysing wound closure or gene expression, probably due to the high cytotoxicity effects of other components in the gel. Thus, our data point out that CHX is highly efficient in blocking bacteria growth, but it shows high cytotoxicity on gingival fibroblasts causing cell death.

Gel C (Lacer Mucorepair Gel) contains enoxolone (or glycyrrhetinic acid), which is a classic type of anti-inflammatory agent with an antibacterial effect, involved in regulating the immune function of the skin, eliminating inflammation, preventing allergies and cleaning the skin [8]. This component is responsible for the inhibition of the bacterial growth in vitro [28,29] and the inhibition of fibroblast growth with associated morphological changes [30]. In addition, this gel has asiaticoside that is commonly used as a treatment for wounds and burns due to its regenerative role stimulating the synthesis of collagen type 1 in human fibroblasts [20]. However, asiaticoside has been shown to have an antiproliferative and toxic effect on cells [31] and to induce apoptosis in human breast cancer cells [32]. These explains the results obtained in the present study, with a demonstrated antibacterial action and a high cytotoxicity on gingival cells, in spite of including hyaluronic acid 0.2% in its formulation.

Continuing with the other gels evaluated, the strong cytotoxic and the high inhibition of *P. gingivalis* growth rate of Gel F (Oralsan NBF Gel Gengivale) might be attributed to propolis. This component produces adverse effects on the proliferation of human fibroblasts and a decrease of living cells, with changes in the cell morphology from regular and spindle-shaped forms [33]. Propolis has also potent antimicrobial activity against *P. gingivalis* [34]. Furthermore, it also contains eucalyptus globulus oil that has been shown to have antibacterial effects against a variety of bacteria [35]. This gel also contains vitamin C and E, both accelerating gingival wound healing and preventing gingival bleeding in preclinical in vivo studies [23,36]. But the present in vitro study has not been able to demonstrate any regenerative properties.

Gel G (Klirich Pro) also caused a high cytotoxicity on GF and a strong antimicrobial activity. Thus, these findings could be presumably caused by CPC, this molecule’s positive charge facilitates binding to negatively charged bacterial surfaces and, consequently, its antimicrobial activity. Moreover, CPC is effective in preventing dental plaque and in reducing gingivitis [37]. Furthermore, the FDA has considered concentration of 0.1% CPC as safe for short term use [38]. CPC, however, has exhibited potent in vitro cytotoxicity effect on fibroblasts at concentrations higher than 0.003% [39] and potent antimicrobial properties [39]. This gel also contains eugenol, a hydroxyphenyl propene that has antibacterial activity [9]. However, it has been shown to have a cytotoxic effect against human gingival fibroblasts and endothelial cells [40] and antimicrobial activity against fungi and a wide range of gram-negative and gram-positive bacteria [9,41].

Gel H (Synthos Biogel) contains chondroitin sulfate, which has been shown to be involved in fibroblast cell migration and to favour wound closure [21]. In addition, it also contains CHX but in a low concentration (0.025%), producing a lower inhibition of bacterial growth but without being cytotoxic on GF. Nevertheless, this study has not been able to demonstrate that it has regenerative properties based on wound closure and gene expression results, since it has not shown superior results compared to the control group. These results show that despite presenting satisfactory biocompatibility, it does not sufficiently stimulate the expression of the genes necessary for proper healing and regeneration.

Gel I (EMD) consists of the amelogenin fraction of porcine enamel matrix suspended in a vehicle of PGA [22]. Amelogenins have the ability to bind to extracellular matrix proteins and regulate their adhesive properties, as well as to stimulate the proliferation and migration of GF [42], which confirms and explains the regenerative effect presented in the wound closure after 48 h and gene expression results on ECM markers. PGA is a propylene glycol ester of alginic acid, which is commonly used in food and pharmaceuticals as a thickening agent [43]. Other studies revealed that PGA has antimicrobial properties, being a common ingredient in various aerosol disinfectants [44]. Although the antibacterial mechanism of PGA is unknown, it is likely that is due to its ability to dehydrate the bacterial cell membrane, being an alcohol; or it is related to its acidic pH, since PGA has a low pH of about 5.0 [22,44]. EMD was also reported to suppress the growth of *P. gingivalis* at a concentration of more than 5 mg/mL [45]. This explains why in this study there is no inhibition of *P. gingivalis* growth rate, because we have used a lower concentration of EMD (1.5 mg/mL).

Regarding the effects of EMD on the levels of mRNA expression of the different genes, this study shows that treatment with EMD increases the expression of the COL1A1, COL3A1, TGF-β1, ACTA2, END, MMP-1 and TIMP-1 genes, while it downregulates the expression of DCN. The progression of periodontitis is characterized by an increase in the collagen degradation, producing a loss of connective tissue [46]. Thus, it was interesting to find an increased expression of the COL1A1 and COL3A1 genes, which are associated with the production of type I collagen and type III collagen, respectively, and associated with increased differentiation of human gingival fibroblasts and scarless wound healing [47]. DCN, a small leucine-rich proteoglycan highly expressed in human gingiva that regulates fibril organization of collagen, including type I and type III [48], was downregulated after EMD treatment. These results can be explained by the fact that a decrease in decorin expression has been related with an inverse association with cell proliferation in regenerating gingival fibroblasts, while is upregulated when cells reach quiescence or are subjected to growth inhibition [49].

ECM undergoes constant changes in its components, mainly due to collagen degradation mediated by MMP-1 [47], leading to accelerated degradation of ECM if there is an excess of its activity [50]. To regulate this process is the inhibitor TIMP-1, which controls and neutralizes the activity of MMP-1 through proteolysis [51]. In this case, EMD has significantly increased the expression levels of MMP-1 and its inhibitor TIMP-1, which could be indicative of an increase in the ECM remodeling (formation/degradation), due to the regenerative properties of amelogenins [42,52].

The characteristics of wound healing also depend on the response of fibroblasts to produce and organize the components of ECM in proportional amounts and according to a healthy tissue [47]. TGF-β1 is the main activator of the expression of collagen and other components of ECM [46]; however, accumulation of TGF-B1 and END after tissue injury, causes the differentiation of fibroblasts into myofibroblasts, which express contractile proteins such as ACTA2, contributing to tissue repair during wound healing, but, if over-expressed, can lead to fibrogenic conditions [53]. In this study, it was observed that in the cells treated with EMD, the expression of TGF-β1, ACTA2 and END was increased compared to control, which could be related to tissue repair during wound healing.

In the 3D model, we only tested Synthos Biogel and EMD, which did not cause a deleterious effect on the GTE, similar to the 2D gingival fibroblast model. This 3D study shows that treatment with EMD increases the release of MMP-1 and TIMP-1 with respect to the control, in accordance to the 2D model. Thus, EMD could facilitate tissue regeneration through the activation of the collagenase MMP-1, that degrades matrix proteins, and it inhibitor TIMP-1 [54]. Moreover, EMD increased expression of IL-6 [55,56], which besides being considered a classic inflammatory cytokine, it has also many regenerative and anti-inflammatory activities and have a crucial role during wound healing [57]. EMD and Synthos Biogel also increased the release of IL-4, which is a pleiotropic cytokine with an important role in wound healing since it induces the production of components of ECM such as type I and type III collagen and stimulates the proliferation of fibroblasts [57]. Immunostaining results of the 3D model after application of EMD or Synthos Biogel, confirmed the maintenance of a good multilayer epithelial structure in which layers are organized similarly to the cells in native oral mucosa and that shows expression of different markers typical of gingival tissue. It has been observed that the collagen layer provides a support that allows the growth and development of human fibroblasts, because these cells express the mesenchymal vimentin marker [58]. We also observed that all groups treated presented a well differentiated epidermis as revealed by involucrin expression, a constituent of the cornified envelope during terminal differentiation [59]. Positive IHC staining for keratin 17 was observed in the epidermis, which is known to be expressed in the basal layer of complex epithelia [60]. Furthermore, keratinocytes expressed keratin 19, a basal marker of differentiation and proliferating epithelial cells [61,62], and Ki-67 a proliferation marker [63]. The detection of keratin 19 and Ki-67 in this GTE demonstrated the proliferative capacity of the cells. Qualitatively, we observed no differences among the groups studied, suggesting no major pathological changes in the of human tissue equivalent of gingiva after the treatments with EMD and Synthos Biogel.

## 5. Conclusions

In conclusion, among all the tested gels, EMD was the one with the best biocompatibility and regenerative properties on gingival fibroblasts but with limited antimicrobial activity on *P. gingivalis* at the conditions tested. On the other hand, gels that showed antimicrobial activity had high cytotoxicity levels. Thus, it is necessary to formulate a gel that has antimicrobial, anti-inflammatory and regenerative properties to treat periodontal disease effectively.

## Figures and Tables

**Table 1:** Different periodontal gels used in this study with the antibacterial and regenerative components. (CHX: chlorhexidine, CPC: cetylpyridinium chloride; PGA: propylene glycol alginate).

Nomenclature	Gel	Manufacturer	Antibacterial Factor	Regenerative Factor
Gel A	Bexident post Gel	ISDIN, Barcelona, Spain	CHX 0.2%	Chitosan 0.5%
Gel B	Perio-Aid Gel Bioadhesive	Dentaid SL, Barcelona, Spain	CHX 0.2%	Hyaluronic acid 0.2%
Gel C	Lacer Mucorepair Gel	Lacer SA, Barcelona, Spain	Enoxolone 0.2%	Hyaluronic acid 0.2%, asiaticoside 0.3%
Gel D	Clorhexidina Lacer Gel Bioadhesive	Lacer SA, Barcelona, Spain	CHX 0.2%	-
Gel E	Oralsan Gel Gengivale	IDS Spa, Savona, Italy	CHX 0.5%	Aloe vera
Gel F	Oralsan NBF Gel Gengivale	IDS Spa, Savona, Italy	Propolis extract, Plant extracts	Propolis extract, Vitamin C and E, Plant extracts
Gel G	Klirich Pro	Itena Clinical, Villepinte, France	CPC 1–2.5%, Eugenol 0–1%, Plant extracts	Hyaluronic acid, Plant extracts
Gel H	Syntoss Biogel	DSI dental solution Israel Ltd., Ashdod, Israel	CHX 0.025%	Chondroitin Sulfate
Gel I	Emdogain (EMD)	Straumann, Basel, Switzerland	PGA	Amelogenin 30 mg/mL

**Table 2:** Genes and primers used in gene expression analysis. Sequence of sense (S) and antisense (A) primers used in the real-time RT-PCR of reference and target genes. Base pairs (bp).

Related Function	Gene	Primer Sequence (5′-3′)	Product Size (bp)	GenBank ID
ECM component	Collagen I α1 (COL1A1)	S: CCTGACGCACGGCCAAGAGG A: GGCAGGGCTCGGGTTTCCAC	122	NM_000088.3
	Collagen III α1 (COL3A1)	S: GGCTACTGGGCCTGGTGGT A: CCACGTTCACCAGGGGCACC	190	NM_000090.3
	Decorin (DCN)	S: ATCTCAGCTTTGAGGGCTCC A: GCCTCTCTGTTGAAACGGTC	146	NM_001920.3
ECM turnover	Matrix metalloproteinase-1 (MMP-1)	S: TGTCAGGGGAGATCATCGGGAC A: TGGCCGAGTTATGAGCTGCA	177	NM_002421.3
	Tissue inhibitor of metalloproteinase-1 (TIMP-1)	S: TTCCGACCTCGTCATCAGGG A: TAGACGAACCGGATGTCAGC	144	NM_003254.2
Wound Healing/Fibrogenic	α-Smooth muscle actin 2 (ACTA2)	S: TAAGACGGGAATCCTGTGAAGC A: TGTCCCATTCCCACCATCAC	184	NM_001141945.1
	Transforming growth factor-β1 (TGF-β1)	S: TGTCACCGGAGTTGTGCGGC A: GGCCGGTAGTGAACCCGTTG	131	NM_000660.4
	Endothelin-1 (END)	S: ACGGCGGGGAGAAACCCACT A: ACGGAACAACGTGCTCGGGA	147	NM_001955.4
Reference gene	Glyceraldehyde-3-phosphate dehydrogenase (GAPDH)	S: TGC ACC ACC AAC TGC TTA GC A: AAG GGA CTT CCT GTA ACA A	87	NM_002046.3
	Beta-Actin (ACTBL2)	S: CTG GAA CGG TGA AGG TGA CA A: AAG GGA CTT CCT GTA ACA A	140	NM_001101.3
	18S ribosomal RNA (18S rRNA)	S: GTAACCCGTTGAACCCCATT A: CCATCCAATCGGTAGTAGCG	151	NR_146156.1

**Table 3:** Primary Antibodies Used for Immunohistochemical Staining.

Antibody	Dilution	Clone	Isotype	Manufacturer
Keratin 17	1:50	E-4	IgG1	Santa Cruz Biotechnology, Inc., Santa Cruz, CA
Keratin 19	1:50	A-3	IgG1	Santa Cruz Biotechnology
Involucrin	1:20	SY5	IgG1	Santa Cruz Biotechnology
Vimentin	1:1000	E-5	IgG1	Santa Cruz Biotechnology
KI-67	1:100	MM1	IgG1	Novocastra, Newcastle, UK

**Table 4:** In vitro results of the commercial gels obtained in this study. (+++) High effect, (++) Moderate effect, (+) Low effect, (−) Null effect and (ND) No determined.

Nomenclature	Gel	Antimicrobial Activity		Cytotoxicity		Wound Closure (2D Model)
		Inhibition of Bacterial Growth	Gingipain Activity	LDH (2D Model)	MTT (3D Model)	
Gel A	Bexident post Gel	+++	+	+++	ND	−
Gel B	Perio-Aid Gel Bioadhesive	+++	+	+++	ND	−
Gel C	Lacer Mucorepair Gel	+++	+	+++	ND	−
Gel D	Clorhexidina Lacer Gel Bioadhesive	+++	+	+++	ND	−
Gel E	Oralsan Gel Gengivale	+++	++	+++	ND	−
Gel F	Oralsan NBF Gel Gengivale	+++	+	+++	ND	−
Gel G	Klirich Pro	+++	++	+++	ND	−
Gel H	Syntoss Biogel	−	+	−	−	−
Gel I	Emdogain (EMD)	−	++	−	−	+

## Data Availability

The data presented in this study are available on request from the corresponding author.
